# 

**Published:** 1834-10-01

**Authors:** 


### CONTENTS

OF THE

### MEDICAL QUARTERLY REVIEW,

### No. V. OCTOBER 1, 1834.

ADDRESS . . . . . . . 1

I.

On the Anatomy and Diseases of the Neck of the Bladder, and of the Urethra: being the Substance of the Lectures delivered in the Theatre of the Royal College of Surgeons, in the year 1830, and in the Westminster Hospital, in 1833 and 1834, by J. G. GUTHRIE, F.R.S. . 3

II.

A Practical Treatise on Diseases of the Uterus and its Appendages. Translated from the French of Mme. VEUVE BOIVIN and A. DUGÈS: with copious Notes, by G. O. HEMING, F.L.S. . . 33

III.

The Physiology, Pathology, and Treatment of Asphyxia; including suspended Animation in New-born Children, and from Drowning, Hanging, Wounds of the Chest, Mechanical Obstructions of the Air-passages, Respiration of Gases, Death from Cold, &c. By JAMES P. KAY, M.D. 46

IV.

Principles and Illustrations of Morbid Anatomy; adapted to the Elements of M. ANDRAL, and to the Cyclopædia of Practical Medicine, &c. By J. HOPE, M.D., F.R.S. . . . . . . 58

V.

Some Observations on the Preparation and Medicinal Employment of the Ioduret and Hydriodate of Iron. By ANTHONY TODD THOMSON, M.D. 60

VI.

Illustrations of the Effects of Poisons. By GEORGE LEITH ROUPELL, M.D. The Plates from original Drawings by ANDREW MELVILLE M'WHINNIE, M.R.C.S. Part II. . . . . . 67

VII.

An Inquiry into the Nature of Sleep and Death, with a View to ascertain the more immediate Causes of Death, and the better Regulation of the Means of obviating them. By A. P. W. PHILIP, M.D. F.R.S. L. & E. . 70

VIII.

Vindication of the pre-eminent Efficacy of Manual Operations in the Cure of Rheumatic and Nervous Diseases, against the malignant and unprincipled Attempts of certain Members of the Medical Profession to decry and obstruct the Practice. By WM. BALFOUR, M.D. L.R.C.S. . 100

*Reviews continued.*

IX.

A Practical Treatise on Medical Jurisprudence, with so much of Anatomy, Physiology, Pathology, and the Practice of Medicine and Surgery, as are essential to be known by Members of Parliament, Lawyers, Coroners, Magistrates, &c. By J. CHITTY, Esq. . . 104

X.

A New View of the Nature of Inflammation, with Cases of Croup and Bronchitis, illustrating a simple and successful Mode of Treatment, and of the Use of the Thymus Gland, &c. By WM. FORRESTER BOW, M.D. 109

XI.

The Dublin Practice of Midwifery. By HENRY MAUNSELL, M.D. . 113

XII.

Traité, Théorique et Pratique, des Blessures par Armes de Guerre; rédigé d'après les Leçons Cliniques de M. le BARON DUPUYTREN; et publié sous sa direction par MM. les Docteurs A. PAILLARD et MARX . 120

XIII.

Nouveau Système de Chimie Organique, fondé sur des Méthodes Nouvelles d'Observation, par F. V. RASPAIL.

A New System of Organic Chemistry, translated from the French of F. V. RASPAIL. With Notes and Additions, by WM. HENDERSON, M.D. . 135

XIV.

A Demonstration of the Nerves of the Human Body. By JOSEPH SWAN 140

XV.

Practical Hints on the Treatment of several Diseases. By JOHN PEACOCK, M.D. . . . . . 143

XVI.

Fragmenta de Viribus Medicamentorum positivis, sive in sano Corpore humano observatis. A SAMUELE HAHNEMANN, M.D. Edidit F. F. QUIN, M.D. . . . . . . 149

XVII.

Cases of Tic Douloureux, and other Forms of Neuralgia. By J. SCOTT, Esq. 152

XVIII.

A Dictionary of Terms employed by the French, in Anatomy, Physiology, Pathology, Practical Medicine, Surgery, Midwifery, Pharmacy, Medical Zoology, Botany, and Chemistry, &c. By SHIRLEY PALMER, M.D. . 156

XIX.

The Jamaica Physical Journal. Edited by JAMES PAUL, Esq. M.R.C.S. . 159

XX.

The Anatomy of the Bones, Joints, and Muscles, exhibiting the Parts as they appear on Dissection, and more particularly in the living Figure; as applicable to the Fine Arts. Second Edition, in two Parts. By GEORGE SIMPSON, M.R.C.S. . . . . 161

XXI.

Dispensary Abuses. Addressed to the Profession . . 163

### Original Communications.

I.

Cases extracted from the Note-book of HENRY DAVIES, M.D. . 166

II.

An Account of the Examination of two Bodies, found in the Vaults of the Ruins of Wymondham Abbey, in Norfolk. By JOHN DALRYMPLE, Esq. [*With an Engraving.*] . . . . 169

III.

Some Account of two Cases of Inflammatory Tumour, produced by the Deposit of the Larva of a large Fly (Œstrus Humanus) beneath the Cutis, in the Human Subject: accompanied with *Drawings* of the Larva. By JOHN HOWSHIP, Esq. . . . . 174

IV.

Account of a Case of Choleroid Affection, produced by the Poison of Muscles. Communicated to the Harveian Society, by THEOPHILUS THOMPSON, M.D. . . . . . 179

V.

Account of Three Cases of Carditis: with Remarks. Communicated to the Harveian Society, by WILLIAM STROUD, M.D. . . 187

VI.

A Case of Delirium Tremens successfully treated by large Doses of Opium. By C. J. ROBERTS, M.D. . . . . 198

### COLLECTANEA.

### PATHOLOGY AND PRACTICE.

Operation for Imperforate Anus . . . . 202

The Remote or Predisposing Causes of Caries of the Teeth . ib.

Insensibility of the Eye . . . . 204

Suture of the Perineum . . . . 205

Method of making the Incision in Extraction of the Cataract . ib.

Cases of the Admission of Air into the Veins . . 206

Septan Ague? . . . . . 209

Case of a Wound of the Trachea and Œsophagus, in which the Hemorrhage stopped without the assistance of Art. By Dr. A. NEUMANN . ib.

Practical Observations on the Method of reducing Dislocations of the Humerus downwards by the Heel in the Axilla. By G. A. PAOLO CUMANO . . . . . 211

Treatment of Aneurism . . . . 213

Malposition of the Heart . . . . ib.

Additional Cases of Club-foot treated by Division of the Tendo-Achillis 214

Paralysis of the Portio Dura . . . . 216

Crying of the Fœtus in Utero . . . . 217

Treatment of Diarrhœa in Infants . . . 218
Case of Extirpation of a Tumour in the Neck, in which the Carotid Artery and Jugular Vein were tied: with Remarks. By WILLIAM GIBSON, M.D. . . . . . 219
A Case of Ossification of the Muscular Tissue. By DAVID L. ROGERS, M.D. 222
Hydatids of the Kidneys passed by the Urethra . . 224
Case of Amaurosis cured by Strychnine . . . 225

### MISCELLANEOUS.

Physiology of the Fœtus . . . . 226

Quain's Anatomical Plates . . . . 227

A Sturdy Hypochondriac . . . . ib.

Influence of the Moon . . . . 228

Ginseng . . . . . . ib.

Mummies . . . . . . 229

Manufacture of Mummies . . . . 230

An Autopsy by Steam . . . . . ib.

Cotton Mills . . . . . 231

The Studies of a Physician . . . . 232

Oculists in the Seventeenth Century . . . 233

The Pulse in Horses . . . . . 234

Temperament . . . . . ib.

Sarracennia . . . . . . 236

Defects in Medical Education . . . . ib.

Medical Responsibility in France . . . 237

A Case of Rabies in the Horse. By Mr. C. MARSHALL, V.S. . ib.

The Mandrake . . . . . 238

Burial in Honey . . . . . ib.

### INTELLIGENCE.

Evidence on the State of the Medical Profession—

Income of the College of Physicians . . . 239

Disqualifications for the Fellowship . . . 240

Meteorological Register. Notices, &c. . . . 242

